# Analysis and Prediction of Exon Skipping Events from RNA-Seq with Sequence Information Using Rotation Forest

**DOI:** 10.3390/ijms18122691

**Published:** 2017-12-12

**Authors:** Xiuquan Du, Changlin Hu, Yu Yao, Shiwei Sun, Yanping Zhang

**Affiliations:** 1 Key Laboratory of Intelligent Computing and Signal Processing of Ministry of Education, Anhui University, Hefei 230601, China; zhangyp2@gmail.com; 2 Center of Information Support & Assurance Technology, Anhui University, Hefei 230601, China; 3 School of Computer Science and Technology, Anhui University, Hefei 230601, China; huchanglin123@163.com (C.H.); yaoyu_2017@163.com (Y.Y.); sunshiwei123@gmail.com (S.S.)

**Keywords:** exon skipping event, RNA-Seq data, sequence information

## Abstract

In bioinformatics, exon skipping (ES) event prediction is an essential part of alternative splicing (AS) event analysis. Although many methods have been developed to predict ES events, a solution has yet to be found. In this study, given the limitations of machine learning algorithms with RNA-Seq data or genome sequences, a new feature, called RS (RNA-seq and sequence) features, was constructed. These features include RNA-Seq features derived from the RNA-Seq data and sequence features derived from genome sequences. We propose a novel Rotation Forest classifier to predict ES events with the RS features (RotaF-RSES). To validate the efficacy of RotaF-RSES, a dataset from two human tissues was used, and RotaF-RSES achieved an accuracy of 98.4%, a specificity of 99.2%, a sensitivity of 94.1%, and an area under the curve (AUC) of 98.6%. When compared to the other available methods, the results indicate that RotaF-RSES is efficient and can predict ES events with RS features.

## 1. Introduction

The complex and diverse process alternative splicing (AS) involves removing noncoding intronic sequences and remaining exons to generate mature mRNA [1]. AS is generally divided into five basic types according to the process [2]: alternative 5’ splice sites, alternative 3’ splice sites, intron retention event, exon skipping (ES) events, and mutually exclusive exons. Approximately 40% to 60% of AS events in the human are estimated to be ES events [3]. Therefore, ES event prediction has become a research hot spot in bioinformatics [4].

Because experiment methods are costly, labor intensive, and have inherent biases and limited coverage, computational prediction of ES events is becoming increasingly popular. Some studies have demonstrated that two kinds of data can regulate the prediction of ES events: genome sequence information and RNA-Seq data. Many classical models have been constructed for genome sequence information. For instance, Sorek et al. [5] combined seven RNA features to classify ES events and achieved a true positive rate of 50% with a false positive rate of 1.8%. Yeo et al. [6] developed a score-based clustering method to obtain 314 intronic splicing regulatory elements from upstream intronic and downstream intronic regions. These results demonstrate that intronic splicing regulatory elements are crucial building blocks for understanding AS regulation, and biological pathways and functions. Chen et al. [7] used a maximum relevance minimum redundancy method to select the optimal feature subset, and then used a quadratic discriminant (QD) function and Bayesian theorem to construct a model with this optimal feature subset. This method obtained an overall accuracy of 68.5%. Dror et al. [8] successfully trained a support vector machine (SVM) using 226 features on the dataset; these features contained 243 ES and 1753 constitutive exons. This method obtained the highest AUC of 0.93 when compared to Neural Network (0.92) and Naïve Bayes (0.89). These methods show that genome sequences provide useful information for ES event prediction. Identifying ES events from RNA-Seq data is also crucial for understanding gene alternative splicing and some human mutation diseases. Many methods have described AS events through features extracted from RNA-Seq data, such as Solas [9], which was built through N_exon_ to read counts on the alternative exon, and predict and quantify alternative isoforms derived solely from exon expression levels. Because Solas only uses N_exon_ and ignores other features associated with ES events, the prediction result of this algorithm contains many false positive samples. Burge et al. [10] developed the PSI (percent spliced in) evaluation method based on N_ni_ that reads counts supporting the inclusive exon and N_ne_ that reads counts supporting the exclusive exon, without considering other features associated with ES events. Similar to Solas, the prediction results of PSI contain many false positive samples. To detect differential alternative splicing events from RNA-Seq data, Shen et al. [11] constructed a Bayesian statistical framework based on N_exon_, N_ni_, N_ne_, N_up intron_ that reads counts on upstream introns, and N_down intron_ that reads counts on downstream introns. The framework obtained a high real-time polymerase chain reaction (RT-PCR) validation rate of 86% for differential ES events. Katz et al. [12] proposed an improved method called MISO (mixture-of-isoforms) based on PSI. The features of MISO not only contain N_ni_ and N_ne_, but also N_exon_, N_up intron_, and N_down intron_. To predict exon splicing, MISO used a Bayesian probabilistic model by calculating the different exon splicing conditions and retaining the posterior probability. Compared to the other methods, MISO results contain the least false positive samples, demonstrating that these new features accurately predict AS events. In addition, a comprehensive review of AS event prediction methods from RNA-Seq data was completed [13] and Feng et al. [14] listed some methods for ES event prediction in cancer with RNA-Seq. Although these methods have achieved good results, some limitations remain.

The slow updating of genome sequences results in lower ES event prediction. For RNA-Seq data, some special RNA expression conditions lead to lower ES event prediction. In addition, the features extracted from single data may have some noise caused by the incompleteness of these data, and these noises may produce unexpected results. Therefore, a method that can reduce the data error and improve accuracy is urgently needed for ES event prediction.

To solve the above-mentioned problems and find more features to describe the ES events, we were inspired by predicting protein complexes in protein-protein interaction networks through multiple information sources [15]. We propose a novel method, RotaF-RSES, to predict the ES event based on Rotation Forest with RS features derived from genome sequence and RNA-Seq data.

## 2. Results

### 2.1. The RotaF-RSES Framework

**Figure 1:**
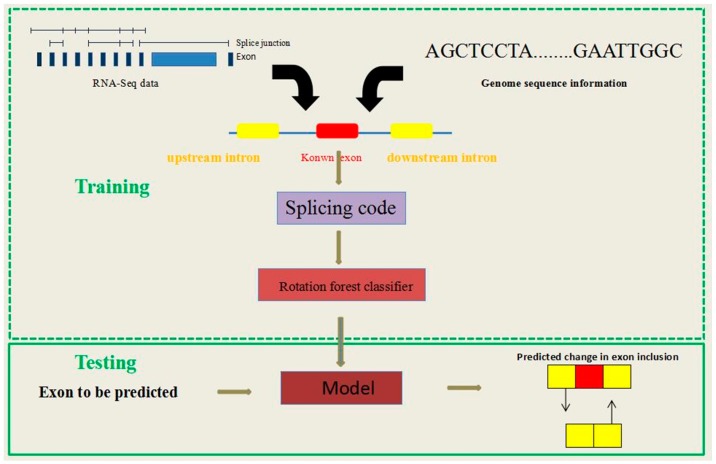
The framework of Rotation Forest classifier to predict ES events with RS features (RotaF-RSES), showing both the training and testing stages. RotaF-RSES involves two steps. Step 1: Obtaining known exons, their upstream and downstream introns, and then extract RNA-Seq features and sequence features according to their RNA-Seq data and sequence information. The above two features, called RS features, were used to build a classification model based on a Rotating Forest algorithm (RotaF-RSES). Step 2: After obtaining the RS features of an unknown type of exon, the RotaF-RSES model was used to determine the type of exon.

RotaF-RSES is a novel method using a Rotation Forest algorithm to facilitate better analysis and ES event prediction with RNA-Seq data and genome sequence information. The method involves the following two main steps (Figure 1).

Firstly, according to the known exon, we extracted the RS features from the RNA-Seq data and genome sequence. After that, these features were used to train the model based on the Rotation Forest.

Secondly, the new exon was sent to the classifier to determine whether the exon is an ES event.

### 2.2. Comparison of Different Features with Random Forest

Some studies have demonstrated that ES event prediction can be regulated through genome sequences or RNA-Seq data. Given their drawbacks mentioned above, we think that if genome sequence information is combined with the RNA-Seq data, better prediction results may be obtained compared to genome sequence or RNA-Seq data. To validate this idea and explore the effects of different data on ES event prediction, some experiments with different features were organized. Because the Random Forest algorithm [16] has been widely adopted in the field of biology and achieves satisfactory results [17], Random Forest was chosen in this study for decision-making.

These experiments were constructed based on the initial features using Random Forest (RF-IFES), equilibrium features using Random Forest (RF-EFES), RNA-Seq features using Random Forest (RF-RFES), sequence features using Random Forest (RF-SFES), and RS features using Random Forest (RF-RSES), with their optimal parameters. The optimal parameters were the same as in the original paper [16], and 100 trees with 9 seeds for RNA-Seq features, 100 trees with 5 seeds for sequence features, 100 trees with 15 seeds for RS feature were used. Table 1 shows the performance comparison of the different features with Random Forest.

From Table 1, the RF-RSES obtains the highest scores for all metrics except specificity and AUC. RF-RSES obtains the highest accuracy, at 96.7%, which is higher by 0.3%, 0.5%, 0.6%, and 14% than RF-IFES (96.4%), RF-EFES (96.2%), RF-RFES (96.1%), and RF-SFES (82.7%), respectively. RF-RSES obtains the highest sensitivity, at 92.2%, which is higher by 3.7%, 5.4%, 2%, and 74.6% than RF-IFES (88.5%), RF-EFES (86.8%), RF-RFES (90.2%), and RF-SFES (17.6%), respectively. RF-IFES and RF-EFES obtain the highest specificity at 98.0%. The specificities of RF-RSES, RF-SFES, and RF-RFES are 97.6%, 95.7%, and 97.3%, respectively. For AUC, RF-RFES and RF-EFES obtain the highest value at 99.3%. However, the AUC of RF-SFES is the lowest at 62.8%.

For the RF-SFES method, we analyzed the results. The test data contained 51 ES events and 255 non-ES events. The RF-SFES predicted 9 true ES events and 11 false ES events, 244 true non-ES events and 42 false non-ES events. The RF-SFES were initially disappointing, but then the results of RF-RSES were combined with RNA-Seq features and sequence features. From these results, RF-RSES had the best overall ability to predict the ES event compared to RNA-Seq data or genome sequence. This result validates our idea.

**Figure 2:**
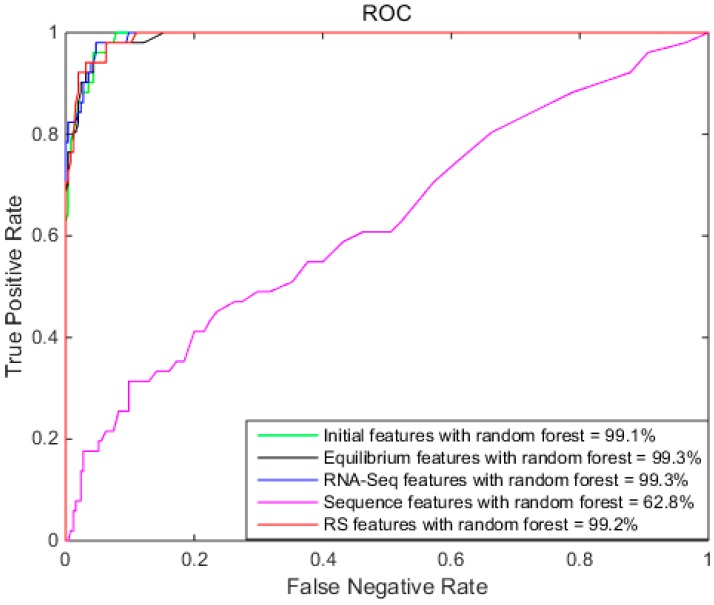
The receiver operating curves (ROC) of different features with Random Forest, showing the initial features with Random Forest (area under a curve (AUC): 99.1%), equilibrium features with Random Forest (AUC: 99.3%), RNA-Seq features with Random Forest (AUC: 99.3%), sequence features with Random Forest (AUC: 62.8%), and RS features with Random Forest (AUC: 99.2%).

Due to the effect of different random decision values, ROC provides a reliable performance comparison. Therefore, Figure 2 shows the ROCs of different features with Random Forest. As shown in Figure 2, the AUC of RF-IFES, RF-EFES, RF-RFES, and RF-RSES are similar. The AUC of RF-SFES was the smallest.

### 2.3. Comparison of Different Algorithms

Although Random Forest was used to perform the predictions in the previous section and achieved good results, we wanted to investigate other machine learning algorithms with RS features. We carefully analyzed and compared other traditional machine learning methods including Random Tree, Naïve Bayes, Bayes Net [18], Naïve Bayes Simple [19], Multilayer Perceptron, Radial Basis Function (RBF) network [20], SVM [21], J48 and Rotation Forest with RS features.

In this work, all classification algorithms were derived in the Waikato environment (WEKA) [22]. The Random Forest contained 100 trees with 15 seeds. SVM uses optimization parameters (c = 2.0, g = 0.001220703125). The Rotation Forest contained 21 seeds. The parameters of other algorithms have default values. Table 2 compares the results of the different algorithms with RS features. The Rotation Forest achieved the best result among all algorithms with an accuracy of 98.4%, a specificity of 99.2%, a sensitivity of 94.1%, and an AUC of 98.6%.

As seen in Table 2, the performance of our Rotation Forest method was higher than any other classifier for RS features, with averages of 12% accuracy, 9% specificity, and 26% sensitivity. The accuracy of Random Forest, Random Tree, Bayes Net, Multilayer Perceptron, J48, and our method were all over 90%. However, the accuracy of Naïve Bayes was only 51.9%. The specificity of almost all the classification algorithms was over 90%, except for Naïve Bayes Simple at 82.8% and Naïve Bayes at 44.7%. The sensitivity of Random Forest, Bayes Net, and our method were over 90%. However, the sensitivity of SVM was 2%. For the AUC, Random Forest was the best with 99.2%, and our method ranked second among all algorithms with 98.6%. The SVM had the smallest AUC with 51.0%. Overall, our method achieved the best result.

We analyzed the experimental results of Naïve Bayes and SVM. The test data included 51 ES events and 255 non-ES events. Naïve Bayes predicted 45 true ES events and 141 false ES events, 114 true non-ES events and 6 false non-ES events. SVM predicted 1 true ES event, 255 true non-ES events, and 50 false non-ES events. 

**Figure 3:**
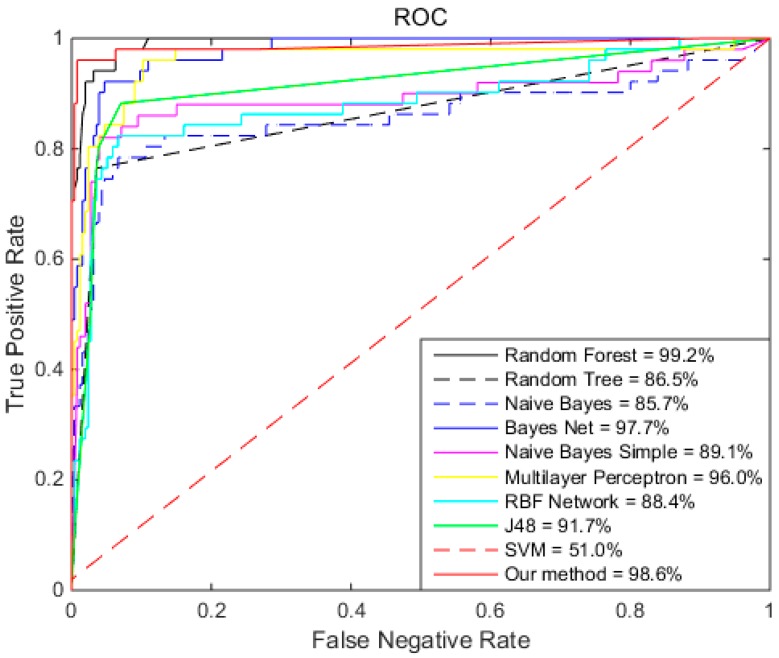
The ROCs of different algorithms on RS features.

To further illustrate the performance of different algorithms, Figure 3 shows the ROCs of different algorithms with RS features. From Figure 3, Rotation Forest has the good AUC, showing that Rotation Forest is the most suitable for ES event prediction.

### 2.4. Comparison of Different Features with Rotation Forest

According to the analysis in the previous section, RotaF-RSES was found to be more suitable for ES event prediction. However, we did not know if the initial features of Rotation Forest (RotaF-IFES), equilibrium features in Rotation Forest (RotaF-EFES), RNA-Seq features with Rotation Forest (RotaF-RFES), or the sequence features with Rotation Forest (RotaF-SFES) could obtain better results than RotaF-RSES. To validate this idea, some experiments were performed with different features of the Rotation Forest algorithm. In this experiment, Rotation Forest contained 21 seeds. Table 3 compares the results of different features of Rotation Forest.

Table 3 shows that the performance of our method was higher than any of the other classifiers. RotaF-RSES obtained the highest accuracy with 98.4%; similar results were obtained for RotaF-EFES (96.7%) and RotaF-RFES (97.4%). RotaF-RSES had the highest specificity at 99.2%, which was 1.2%, 1.2%, 1.2%, and 0.8% higher than RotaF-IFES (98.0%), RotaF-EFES (98.0%), RotaF-RFES (98.0%), and RotaF-SFES (98.4%), respectively. RotaF-RSES had the highest sensitivity with 94.1%, which was 9.8%, 3.9%, 2%, and 86.2% higher than RotaF-IFES (84.3%), RotaF-EFES (90.2%), RotaF-RFES (92.1%), and RotaF-SFES (7.9%), respectively. The same AUC was obtained by RotaF-IFES (98.6%), RotaF-EFES (98.6%), and RotaF-RSES (98.6%). The AUC for RotaF-RFES and RotaF-SFES were 98.3% and 62.3%, respectively. For RotaF-SFES, the sensitivity was only 7.9%, that of RotaF-RFES was 92.1%, and that of RotaF-RSES was 94.1%, so the sequence features improved ES event prediction. As RotaF-RSES had the best performance, we used the RS features with Rotation Forest to build our model for ES event prediction.

**Figure 4:**
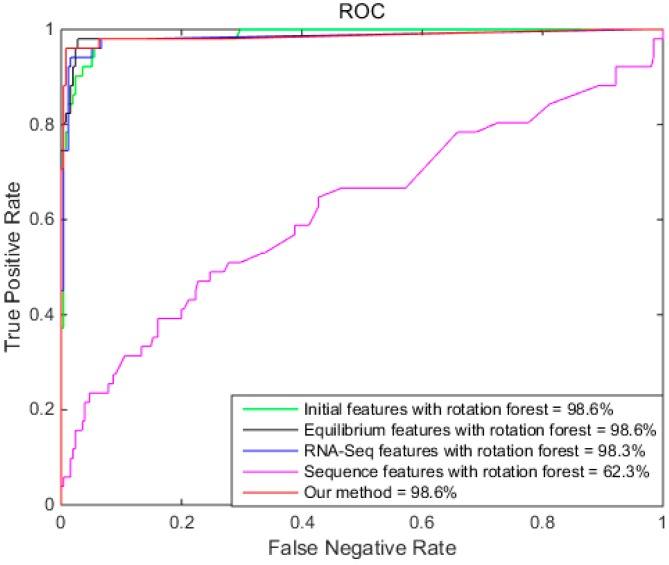
The ROCs of different features with Rotation Forest.

To further illustrate the performance of different features of Rotation Forest, Figure 4 shows the ROCs of different features of Rotation Forest. Figure 4 demonstrates that the AUC of RotaF-IFES, RotaF-EFES, RotaF-RFES, and our method are similar, and that the RotaF-EFES and our method performed the best. The AUC of RotaF-SFES was the smallest.

### 2.5. Comparing RotaF-RSES with Other Methods

We first compared RotaF-RSES with the state-of-the-art method ESFinder (a Random Forest classifier to identify ES events from RNA-Seq data) [16]. ESFinder predicts ES events using Random Forest with RNA-Seq data. As shown in Table 4, RotaF-RSES outperforms ESFinder for most metrics. Here, the ESFinder parameters were the same as in the original paper and the parameters of RotaF-RSES contained 21 seeds.

From Table 4, the accuracy of RotaF-RSES was 98.4%, 2.2% higher than ESFinder (96.2%). The specificity of RotaF-RSES was 99.2%, 1.2% higher than ESFinder (98.0%). The sensitivity of RotaF-RSES was 94.1%, 7.3% higher than ESFinder (86.8%). However, the AUC of ESFinder was 99.3%, 0.7% higher than RotaF-RSES (98.6%). Overall, RotaF-RSES outperformed ESFinder, indicating that RotaF-RSES is efficient and can predict ES events.

We investigated why the specificity and sensitivity of RotaF-RSES were higher than ESFinder, but the AUC of ESFinder was higher than RotaF-RSES. To answer the question, we checked their experimental results. The test data contained 51 ES events and 255 non-ES events. ESFinder predicted 46 true ES events and 7 false ES events, 248 true non-ES event and 5 false non-ES events. However, RotaF-RSES predicted 48 true ES events and 3 false ES events, 253 true non-ES event and 2 false non-ES events. As RotaF-RSES had many true non-ES events (253), the AUC of RotaF-RSES is was slightly smaller than that of the ESFinder.

**Figure 5:**
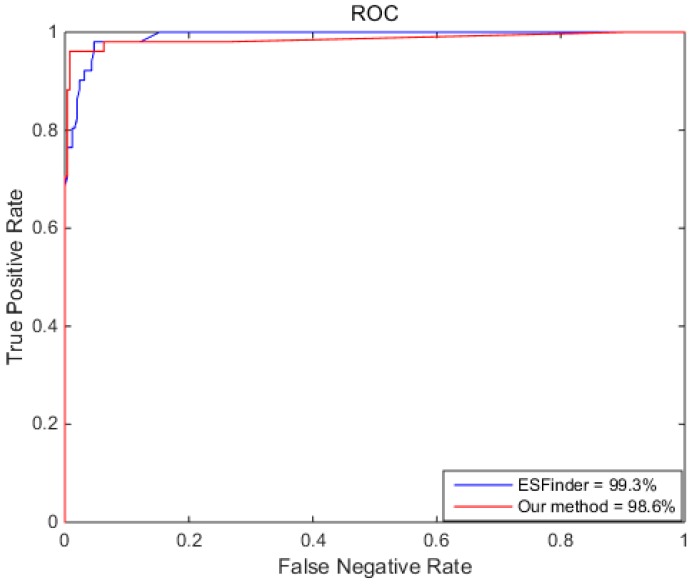
The ROCs of different methods.

To further illustrate the performance of both ESFinder and our method, Figure 5 shows the ROCs of ESFinder and our method. Although the AUC of ESFinder is larger than that of our method, the AUC of our method is greater than ESFinder at the beginning.

**Figure 6:**
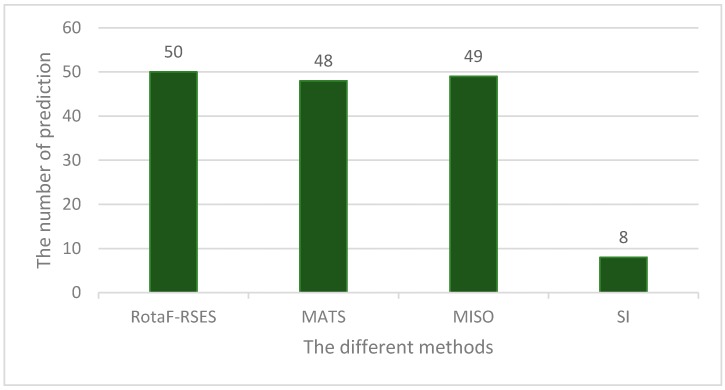
The prediction results of the RotaF-RSES, MATS, MISO, and SI methods on test data.

Next, we compared RotaF-RSES with MATS (multivariate analysis of transcript splicing) [11], MISO [12], and SI (splice index) [23] on the test data. MATS, MISO, and SI are well-known methods for ES event prediction using different read features with RNA-Seq data. The test data contained 51 ES events and 255 non-ES events. As shown in Figure 6, the RotaF-RSES predicted 50 ES events including 2 false ES events, MATS predicted 48 ES events, MISO predicted 49 ES events, and SI predicted 8 ES events.

### 2.6. RotaF-RSES Prediction on Independent Test Data

We constructed independent test data that contained the existing ES events derived from the UCSC Alt events dataset, with a total of 83454 ES event instances. For the skeletal muscle and brain RNA-Seq data, 306 events were used as the test data, and 612 as the training data, so the remaining 82,536 events were used as the independent test data.

To validate our method on the independent test data, we compared RotaF-RSES to ESFinder, MATS, MISO, and SI. The results are shown in Table 5. The number of predictions for RotaF-RSES, ESFinder, MATS, MISO, and SI were 1910, 1977, 91, 140, and 179, respectively.

## 3. Discussion

The classic method to identify ES events involved features derived from genome sequences using a machine learning method [24,25]. With high-throughput technology, extracting features from RNA-Seq data to predict ES events is another popular method [26,27,28]. Although these methods have been reasonably successful, the results could be improved. Predicting ES events with more precision and improving the prediction results based on past research results have been challenging. To address these problems in the present work, a new feature was constructed, called RS features, that consist of RNA-Seq features derived from the RNA-Seq data and sequence features derived from the genome sequence. We simultaneously propose a novel Rotation Forest classifier to predict ES events based on the RS features (RotaF-RSES).

In this work, we observed some relationships between the RS, sequence, and RNA-Seq features. We analyzed the effect of different features on ES event prediction based on Random Forest, and found that the RS features obtained a sensitivity of 92.2%, and the sequence features and RNA-Seq features were the highest at 90.2%, whereas the accuracy, specificity, and AUC were similar. Experimental results showed that the predictive power of the RS features was higher than RNA-Seq features or the single sequence features alone.

Some researchers have shown that different methods have different effects on identifying ES events based on the same features [29,30]. We analyzed the predictive power of the ten most common machine learning methods based on RS features, and found that Rotation Forest obtained the highest sensitivity with 94.1%, 2% higher than Random Forest. To further investigate the relationship between RS features, sequence features, and RNA-Seq features, we analyzed the effect of different features on the prediction of ES events based on Rotation Forest, and obtained the same conclusion as above. Compared to other methods, RS features combined Rotation Forest also obtained relatively good results.

In conclusion, we found that the predictive power of the RS features was higher than the RNA-Seq features or the single sequence features alone. Our experiments showed that RotaF-RSES is an efficient method for ES event prediction. In the future, we will analyze the reason that these sequence features achieve better results when binding RNA-Seq features.

## 4. Materials and Methods

### 4.1. Dataset

To compare our method to the existing state-of-the-art method, we used the same benchmark dataset as ESFinder [16]. It was constructed by the incorporating the predictions of MATS [11], MISO [12], and SI [23]. In this dataset, the instances hit by at least two of three methods are marked as an ES event and those hit by none of the three methods are marked as a non-ES event. The training data contain 102 ES events and 510 non-ES events; the test data contain 51 ES events and 255 non-ES events.

The genome sequences were collected from the UCSC (University of California Santa Cruz, Santa Cruz, CA, USA) Genome Browser Home. Given the strand, the start and end positions of the exon, and the upstream and downstream introns, we accurately obtained sequence information. The RNA-Seq data were collected from human brain (GSM325476) and skeletal muscle (GSM325479). The raw RNA-Seq were mapped by Tophat2 to the genome sequences, then sorted and stored in a BAM file [31]. In addition, HTSeq-count with the intersection-strict standard was used to count in this study [32]. The source code and data of our approach can be used via http://ailab.ahu.edu.cn:8087/RotaF-RSES/index.html.

### 4.2. Feature Extraction

The RS features were composed of RNA-Seq features derived from the RNA-Seq data and sequence features derived from genome sequences. These sequence features were composed of structure features and short motif features. The RNA-Seq features were composed of initial features and equilibrium features. A detailed description is given below.

### 4.3. Sequence Features

The sequence features were composed of structure features and short motif features. Here, structure features included the length of the upstream intron, exon, and downstream intron, which are important for ES event prediction. Generally, the length of the intron is much larger than the adjacent length of exon [33].

The short motif features include single-tuple counts, computed separately for downstream introns, exons, and upstream introns, resulting in a total of 4 × 3 = 12 features. These short motif features have been previously shown to be helpful for ES event prediction [24,34].

### 4.4. RNA-Seq Features

The RNA-Seq features were composed of initial features and equilibrium features. For each RNA-Seq data, the following six basic features were extracted: N_exon_, N_up intron_, N_down intron_, N_ni_, N_ne_, and N_gene_. Table 6 shows a detailed description of these six basic features. The six features BN_exon_, BN_up intron_, BN_down intron_, BN_ni_, BN_ne_, and BN_gene_ were obtained from human brain RNA-Seq data, and the other six features SN_exon_, SN_up intron_, SN_down intron_, SN_ni_, SN_ne_, and SN_gene_ were obtained from human skeletal muscle RNA-Seq data. These 12 features constitute initial features. Table 7 shows a detailed description of these initial features.

The equilibrium features were composed of normalized features, P features, and divergence features. For each RNA-Seq data, the following six basic normalized features were extracted NORM_exon_, NORM_up intron_, NORM_down intron_, NORM_ni_, NORM_ne_, and NORM_gene_. Table 8 shows a detailed description of these six basic normalized features. In the Table 8, L_e_ is the length of alternative exon, L_r_ is the length of RNA-Seq read, o is the length of the anchor [35], T_num_ is the number of the total mapped read in the sample, and L_g_ is the length of gene. L_e_ − L_r_ + 1 is the effective length of exon where reads are mapped; L_r_ + 1 − 2o is the effective length of upstream intron where reads are mapped; L_e_ − L_r_ + 1 + 2 × (L_r_ + 1 − 2o) is the effective length of the inclusive isoform where reads are mapped; and L_g_ − L_r_ + 1 is the effective length of the gene where reads are mapped. Six normalized features were obtained through human brain RNA-Seq data: BNORM_exon_, BNORM_up intron_, BNORM_down intron_, BNORM_ni_, BNORM_ne_, and BNORM_gene_. Another six normalized features were obtained through human skeletal muscle RNA-Seq data: SNORM_exon_, SNORM_up intron_, SNORM_down intron_, SNORM_ni_, SNORM_ne_, and SNORM_gene_. Table 9 shows a detailed description of these 12 normalized features.

Feature P simultaneously describes the percentage of NORM_ni_, defined by Equation (1), that results in two features from brain (BP) and human skeletal muscle (SP) RNA-Seq data. In addition, seven features, named divergence features, that indicate divergence of normalized read count features between skeletal muscle and brain RNA-Seq data were also used: Δ_exon_, Δ_up intron_, Δ_down intron_, Δ_ni_, Δ_ne_, Δ_gene_, and Δ_p_. These seven features are defined by Equations (2) through (8), respectively. In summary, Table 10 describes the equilibrium features.

(1) $$P=\frac{{\mathrm{NORM}}_{\mathrm{ni}}}{{\mathrm{NORM}}_{\mathrm{ni}}+{\mathrm{NORM}}_{\mathrm{ne}}}$$

(2) $$\Delta_{\mathrm{exon}}={\mathrm{BNORM}}_{\mathrm{exon}}-{\mathrm{SNORM}}_{\mathrm{exon}}$$

(3) $$\Delta_{\mathrm{up}\mathrm{intron}}={\mathrm{BNORM}}_{\mathrm{up}\mathrm{intron}}-{\mathrm{SNORM}}_{\mathrm{up}\mathrm{intron}}$$

(4) $$\Delta_{\mathrm{down}\mathrm{intron}}={\mathrm{BNORM}}_{\mathrm{down}\mathrm{intron}}-{\mathrm{SNORM}}_{\mathrm{down}\mathrm{intron}}$$

(5) $$\Delta_{\mathrm{ni}}={\mathrm{BNORM}}_{\mathrm{ni}}-{\mathrm{SNORM}}_{\mathrm{ni}}$$

(6) $$\Delta_{\mathrm{ne}}={\mathrm{BNORM}}_{\mathrm{ne}}-{\mathrm{SNORM}}_{\mathrm{ne}}$$

(7) $$\Delta_{\mathrm{gene}}={\mathrm{BNORM}}_{\mathrm{gene}}-{\mathrm{SNORM}}_{\mathrm{gene}}$$

(8) $$\Delta_{P}=BP-SP$$

### 4.5. Rotation Forest

Rotation Forest [36] is an ensemble learning algorithm based on a decision tree that adopts the concept of feature transformation to improve the accuracy of the base classifiers. Rotation Forest uses features transformation to obtain the feature subspace and reorganize a complete set of attributes by principal components analysis (PCA) [37]. The following is a Rotation Forest training process.

Given initial instances set S(N×D), where N and D is the number of instances and features, respectively:

(1) We split D randomly into K subsets. The feature number of each subset was M(M=D/K), which obtains k instances subsets, based on feature subsets $S_{i}(i=1,2,3.....,k)$.
(2) Using PCA to obtain feature conversion, for example, subset Si and obtain M feature vector, and M’ feature vectors (non-zero) were selected to form a feature vector matrix $a_{i}=[a_{i1},\ldots ,a_{iM}]$.
(3) Step (2) is repeated and the result is inputted into a matrix R. We found these features and their initial position in S according to the feature vector in R. Each feature vector was rearranged according to the initial position to obtain a new R*, and a new sample $S_{\mathrm{new}}=S\times R^{*}$ was set up.
(4) Multiple base classifiers were obtained using the above procedure. The final result was determined by the maximum class confidence.

### 4.6. Performance Evaluation

The prediction of an ES event is a binary classification problem. In this experiment, accuracy, specificity, and sensitivity were chosen to measure the performance of classifiers:

(9) $$\mathrm{Accuracy}=\frac{\mathrm{TP}+\mathrm{TN}}{\mathrm{TP}+\mathrm{TN}+\mathrm{FP}+\mathrm{FN}}$$

(10) $$\mathrm{Specificity}=\frac{\mathrm{TN}}{\mathrm{TN}+\mathrm{FP}}$$

(11) $$\mathrm{Sensitivity}=\frac{\mathrm{TP}}{\mathrm{TP}+\mathrm{FN}}$$

where TP denotes the number of true positive ES events, TN is the number of true negative non-ES events, FP is the number of false positive ES events, and FN is the number of false negative non-ES events. In addition, the receiver operating characteristic (ROC) curve is often used to evaluate classifier performance [38]. A classifier conducts predictions based on a threshold, which is generally defined as 0.5. When the threshold value is changed, new predictions are obtained and a point can be plotted with the true positive rate (TPR) versus the false positive rate (FPR) for different threshold values.

(12) $$\mathrm{TPR}=\frac{\mathrm{TP}}{\mathrm{TP}+\mathrm{FN}}$$

(13) $$\mathrm{FPR}=\frac{\mathrm{FP}}{\mathrm{FP}+\mathrm{TN}}$$

The area under a curve (AUC) for the ROC curve is also used. When the AUC value of a predictor is larger than the area of other ROC curves, the predictor is considered better than other predictors [39]. Because our main goal was to predict ES events, when other metrics were similar, the higher the sensitivity, the better the model.

## 5. Conclusions

In this study, to reduce the error caused by RNA-Seq data or genome sequence on ES event prediction, we propose a novel method named RotaF-RSES, which uses Rotation Forest with RS features composed of RNA-Seq features and sequence features. We explored the effects of two kinds of data on ES event prediction. Five different feature sets derived from the above data were selected for the analysis. The results indicated that RS features are better than any individual dataset for ES event prediction. To investigate the ability of different machine learning algorithms with RS features, ten algorithms were used for comparison and analysis. The results showed that Rotation Forest had the best performance. In addition, we analyzed the impact of different feature sets with Rotation Forest. The above five features were also selected for analysis. The results reinforced our ideas. Finally, to confirm the ability of RotaF-RSES to predict ES events, four methods were used to compare the performance of RotaF-RSES. The results confirmed that RotaF-RSES is efficient and has a strong ability to predict ES events. RotaF-RSES could provide biologists more accurate results for ES event studies.

## Figures and Tables

**Table 1:** Performance comparison of different features with Random Forest.

Features	Accuracy	Specificity	Sensitivity	AUC
Initial	96.4%	98.0%	88.5%	99.1%
Equilibrium	96.2%	98.0%	86.8%	99.3%
RNA-Seq	96.1%	97.3%	90.2%	99.3%
Sequence	82.7%	95.7%	17.6%	62.8%
RS	96.7%	97.6%	92.2%	99.2%

**Table 2:** Performance comparison of different algorithms on RS features.

Algorithm	Accuracy	Specificity	Sensitivity	AUC
Random Forest	96.7%	97.6%	92.2%	99.2%
Random Tree	93.1%	96.5%	76.5%	86.5%
Naïve Bayes	51.9%	44.7%	88.2%	85.7%
Bayes Net	94.1%	94.5%	92.2%	97.7%
Naïve Bayes Simple	84.2%	82.8%	88.0%	89.1%
Multilayer Perceptron	93.1%	97.7%	70.6%	96.0%
RBF network	86.9%	99.6%	23.5%	88.4%
J48	93.1%	96.5%	76.5%	91.7%
SVM	83.7%	100%	2%	51.0%
Our Method	98.4%	99.2%	94.1%	98.6%

**Table 3:** Comparison results of different features with Rotation Forest.

Features	Accuracy	Specificity	Sensitivity	AUC
Initial	95.8% (96.4%)^RF^	98.0% (98.0%)^RF^	84.3% (88.5%)^RF^	98.6% (99.1%)^RF^
Equilibrium	96.7% (96.2%)^RF^	98.0% (98.0%)^RF^	90.2% (86.8%)^RF^	98.6% (99.3%)^RF^
RNA-Seq	97.4% (96.1%)^RF^	98.0% (97.3%)^RF^	92.1% (90.2%)^RF^	98.3% (99.3%)^RF^
Sequence	83.0% (82.7%)^RF^	98.4% (95.7%)^RF^	7.9% (17.6%)^RF^	62.3% (62.8%)^RF^
RS	98.4% (96.7%)^RF^	99.2% (97.6%)^RF^	94.1% (92.2%)^RF^	98.6% (99.2%)^RF^

( )^RF^ is the Random Forest value.

**Table 4:** Performance comparison between ESFinder and our method.

Method	Accuracy	Specificity	Sensitivity	AUC
ESFinder	96.2%	98.0%	86.8%	99.3%
Our method	98.4%	99.2%	94.1%	98.6%

**Table 5:** The predictions of RotaF-RSES, ESFinder, MATS, MISO, and SI for independent test data.

Different Methods	Our Method	ESFinder	MATS	MISO	SI
Correct Predictions	1910	1977	91	140	179

**Table 6:** The description of the six basic features.

Feature	The Description of These Features
$N_{\mathrm{exon}}$	Read counts on exons
$N_{\mathrm{up}\mathrm{intron}}$	Reads counts on the upstream intron
$N_{\mathrm{down}\mathrm{intron}}$	Reads counts on the downstream intron
$N_{\mathrm{ni}}$	Reads counts supporting the inclusive exon
$N_{\mathrm{ne}}$	Reads counts supporting the exclusive exon
$N_{\mathrm{gene}}$	Reads counts on the corresponding gene

**Table 7:** The description of all initial features.

Skeletal Muscle (S)	Brain (B)
${\mathrm{SN}}_{\mathrm{exon}}$	${\mathrm{BN}}_{\mathrm{exon}}$
${\mathrm{SN}}_{\mathrm{up}\mathrm{intron}}$	${\mathrm{BN}}_{\mathrm{up}\mathrm{intron}}$
${\mathrm{SN}}_{\mathrm{down}\mathrm{intron}}$	${\mathrm{BN}}_{\mathrm{down}\mathrm{intron}}$
${\mathrm{SN}}_{\mathrm{ni}}$	${\mathrm{BN}}_{\mathrm{ni}}$
${\mathrm{SN}}_{\mathrm{ne}}$	${\mathrm{BN}}_{\mathrm{ne}}$
${\mathrm{SN}}_{\mathrm{gene}}$	${\mathrm{BN}}_{\mathrm{gene}}$

**Table 8:** The description of basic normalized features.

Feature	The Definition of These Features
${\mathrm{NORM}}_{\mathrm{exon}}$	$N_{\mathrm{exon}}\times \frac{1000000000}{(L_{e}-L_{r}+1)\times T_{\mathrm{num}}}$
${\mathrm{NORM}}_{\mathrm{up}\mathrm{intron}}$	$N_{\mathrm{up}\mathrm{intron}}\times \frac{1000000000}{(L_{r}+1-2o)\times T_{\mathrm{num}}}$
${\mathrm{NORM}}_{\mathrm{down}\mathrm{intron}}$	$N_{\mathrm{down}\mathrm{intron}}\times \frac{1000000000}{(L_{r}+1-2o)\times T_{\mathrm{num}}}$
${\mathrm{NORM}}_{\mathrm{ni}}$	$N_{\mathrm{ni}}\times \frac{1000000000}{(L_{e}-L_{r}+1+2\times (L_{r}+1-2o))\times T_{\mathrm{num}}}$
${\mathrm{NORM}}_{\mathrm{ne}}$	$N_{\mathrm{ne}}\times \frac{1000000000}{(L_{r}+1-2o)\times T_{\mathrm{num}}}$
${\mathrm{NORM}}_{\mathrm{gene}}$	$N_{\mathrm{gene}}\times \frac{1000000000}{(L_{g}-L_{r}+1)\times T_{\mathrm{num}}}$

**Table 9:** The description of the normalized features.

Skeletal Muscle (S)	Brain (B)
${\mathrm{SNORM}}_{\mathrm{exon}}$	${\mathrm{BNORM}}_{\mathrm{exon}}$
${\mathrm{SNORM}}_{\mathrm{up}\mathrm{intron}}$	${\mathrm{BNORM}}_{\mathrm{up}\mathrm{intron}}$
${\mathrm{SNORM}}_{\mathrm{down}\mathrm{intron}}$	${\mathrm{BNORM}}_{\mathrm{down}\mathrm{intron}}$
${\mathrm{SNORM}}_{\mathrm{ni}}$	${\mathrm{BNORM}}_{\mathrm{ni}}$
${\mathrm{SNORM}}_{\mathrm{ne}}$	${\mathrm{BNORM}}_{\mathrm{ne}}$
${\mathrm{SNORM}}_{\mathrm{gene}}$	${\mathrm{BNORM}}_{\mathrm{gene}}$

**Table 10:** The equilibrium features.

Skeletal Muscle (S)	Brain (B)	Divergence
${\mathrm{SNORM}}_{\mathrm{exon}}$	${\mathrm{BNORM}}_{\mathrm{exon}}$	$\Delta_{\mathrm{exon}}$
${\mathrm{SNORM}}_{\mathrm{up}\mathrm{intron}}$	${\mathrm{BNORM}}_{\mathrm{up}\mathrm{intron}}$	$\Delta_{\mathrm{up}\mathrm{intron}}$
${\mathrm{SNORM}}_{\mathrm{down}\mathrm{intron}}$	${\mathrm{BNORM}}_{\mathrm{down}\mathrm{intron}}$	$\Delta_{\mathrm{down}\mathrm{intron}}$
${\mathrm{SNORM}}_{\mathrm{ni}}$	${\mathrm{BNORM}}_{\mathrm{ni}}$	$\Delta_{\mathrm{ni}}$
${\mathrm{SNORM}}_{\mathrm{ne}}$	${\mathrm{BNORM}}_{\mathrm{ne}}$	$\Delta_{\mathrm{ne}}$
${\mathrm{SNORM}}_{\mathrm{gene}}$	${\mathrm{BNORM}}_{\mathrm{gene}}$	$\Delta_{\mathrm{gene}}$
SP	BP	$\Delta_{P}$
